# Prioritising Mentorship: The Key Attributes We Should Focus on From Our Clinical Teaching Fellows in Early Years Medical Education

**DOI:** 10.1111/tct.70053

**Published:** 2025-02-21

**Authors:** Nadia Lascar, Wendy Leadbeater, Noor Al‐Antary, Joseph Cowling, Claire Joanne Stocker

**Affiliations:** ^1^ Aston Medical School Aston University College of Health and Life Sciences Birmingham UK; ^2^ Department of Biomedical Sciences University of Birmingham College of Medicine and Health Birmingham UK; ^3^ University Hospitals Birmingham NHS Foundation Trust Birmingham UK

**Keywords:** clinical teaching fellow, mentoring, medical education, attributes, early years, pastoral care

## Abstract

**Background:**

Clinical teaching fellows (CTFs) enhance medical students' education, yet their role in early undergraduate years is less understood. This study explores the key attributes of CTFs, as perceived by medical students, CTFs and staff, that contribute to the quality of students' learning experiences in the early years of medical school.

**Methods:**

This mixed‐methods study was conducted at a UK medical school, involving surveys and focus groups with 102 early‐year MBChB (Bachelor of Medicine, Bachelor of Surgery) students, five CTFs and 15 staff. Participants identified CTF attributes contributing to students' learning experiences in the first 2 years of the programme. Data were analysed using descriptive statistics and thematic analysis.

**Results:**

The study identified 364 attributes, categorised into 12 themes. Key attributes included helpful/supportive, friendly/approachable and educator/facilitator. Students valued CTFs' approachable nature, while staff emphasised their educational role. Focus groups further highlighted the importance of CTFs' mentorship and pastoral care, interpreting helpful/supportive as sharing personal experiences and career guidance, friendly/approachable as building relatable connections and educator/facilitator as providing academic support.

**Conclusion:**

Our study shows that in the early years of medical education, students value the mentoring skills of CTFs over their academic and clinical expertise. Medical schools should provide formal opportunities for CTFs to enhance their mentoring and pastoral care skills and ensure academic staff support these extended roles, potentially leading to improved student satisfaction and better preparation for clinical years. These insights could inform policy and training programs in medical schools globally, enhancing the overall quality of early medical education.

## 1 Background

After graduation from the MBChB (Bachelor of Medicine, Bachelor of Surgery) Programme in the United Kingdom (UK), graduates typically complete a 2‐year foundation programme. Following this training, over 60% take a break from specialty training, with many pursuing education, research or other alternatives [1]. Among these, clinical teaching fellows (CTFs) are early‐career doctors who complement their clinical expertise with medical education experience, develop professional portfolios and seek better work‐life balance [2].

CTFs primarily facilitate learning through classroom teaching and bedside tutorials, offering opportunities for personal and professional development [3]. Recruitment focuses on educational interest, but training and support vary widely, ranging from structured programs to informal, on‐the‐job learning. Typically, CTF educational training includes workshops, seminars and hands‐on activities designed to familiarise them with teaching methods and educational theory. This training often incorporates case studies, role‐playing exercises and collaborative projects to ensure that CTFs can effectively engage with students. Ongoing support for CTFs can include mentorship programs, online resources and continuous professional development opportunities, helping educators stay updated on best practices and providing necessary tools for effective teaching [1].

The scope of CTF roles has evolved from traditional bedside teaching to include curriculum design, assessment and pedagogical research [4, 5, 6]. Their contributions to clinical years are well‐documented, with students appreciating their teaching due to dedicated time and approachability [7, 8, 9, 10]. However, while their involvement in clinical years has been extensively studied, the role of CTFs in early undergraduate education remains underexplored, despite growing recognition of its potential benefits.

Insights from CTFs themselves highlight their unique position to support not only clinical teaching but also broader aspects of students' personal and professional development [11, 12, 13], owing to their near‐peer status and frequent interactions with students [14]. Near‐peer mentors, who are slightly more advanced than the students they mentor, provide relatable guidance and support. Ratcliff and George [15] argued that students in their first clinical year would benefit significantly from CTF mentorship, as they often experience high levels of stress and decreased confidence when progressing from the early years to their initial clinical placement. This need for early CTF involvement was further confirmed by Hossain et al. [16], who highlighted the necessity of researching the potential benefits of such involvement for first year medical students. While a recent study by Couchman et al. [5] recognised the value of CTFs in bringing near‐peer experience to the early years, their research primarily focused on how CTFs were supported in their role by medical school faculty, rather than the student experience.

We have highlighted a gap in the literature regarding how early years medical students perceive the CTF role and how they feel CTFs contribute to their learning experience. The aim of this study was to explore how medical students perceived the CTF role, how these perceptions aligned with CTFs' views of their own role and the expectations CTFs had of academic staff. Through this analysis, we identified the attributes of the CTF role that positively influenced the learning experience of medical students in the early years of medical education.

## 2 Methods

### 2.1 Study Setting

This study was conducted at a UK medical school in the West Midlands, active since 2018 and enrolling 120 students annually. The school employs CTFs to support the early years (Years 1 and 2) of a 5‐year undergraduate entry MBChB programme, exclusively at the Medical School, with no expectation for them to teach in the local teaching hospital. CTFs in the post have completed their 2‐year foundation programme and primarily facilitate team‐based learning [17] tutorials, in which students engage with the curriculum in small groups. CTFs dedicate at least 9 h per week to assisting with basic sciences and clinical skills, working alongside academic staff to provide a clinical perspective and to support lectures and assessments. Additionally, CTFs serves as personal tutors to a small group of early‐year students.

### 2.2 Study Design

This is a mixed‐methods study evaluating the perceptions of the CTFs' role in the first 2 years of the MBChB programme, through surveys and focus groups. Between June and September 2022, a total of 253 MBChB Year 1 and 2 students, six CTFs and 31 faculty members, were invited to participate to the study.

The surveys used open‐ended questions and explored the experiences and perceptions of the role of the CTF in the first and second year of the MBChB programme with students, CTFs and academic staff providing responses to the following:

1. Three attributes characterising the CTFs' role;
2. How do CTFs contribute to the quality of students' learning experience?

After completing the survey, a subset of participants from each of the three groups took part in focus groups (Appendix S1), which examined the survey responses in greater detail, providing more comprehensive insights into their perceptions of the CTFs' role and the attributes identified during the survey analysis. Qualitative data were analysed using thematic analysis [18, 19].

### 2.3 Rigour

The quality of written responses was ensured through analyst triangulation, with independent coding by analysts from diverse backgrounds. The author team included five academics, three of whom are also clinicians and two with expertise in biomedical sciences, contributing a range of perspectives to the analysis. Additionally, rigour was enhanced through a detailed study description (see Study Setting), and an external audit by a researcher outside the focus group to ensure dependability and confirmability.

### 2.4 Data Analysis

The survey responses were analysed descriptively using SPSS (version 26), with chi‐squared tests assessing CTFs' attributes. Significance was set at *p* < 0.05. Focus group discussions were transcribed and analysed using a thematic framework approach [20]. The research team identified key themes and subthemes from the pooled responses of CTFs, staff and students, which were coded for similarities, contrasts, beneficial topics and notable points.

### 2.5 Ethical Considerations

This study was reviewed and approved May 2022 by the Health and Life Sciences Ethics Committee, Aston University reference #1827.

## 3 Results

Survey data were analysed for 102/253 (40%) students, 5/6 (83%) CTFs and 15/31 (48%) staff. Focus group data were analysed for 12 students, eight staff and six CTFs.

### 3.1 Analysis of CTFs' Attributes

When delineating the perception of the role of CTFs through surveying the students, CTFs and faculty staff, a total of 364 distinct attributes (codes) were identified and subsequently classified into 12 broad categories (themes), illustrated in Table 1.

**TABLE 1:** Attributes defining the CTFs' role. The table lists the key attributes (codes) defined by students, CTFs and staff and grouped in broad categories (themes).

Helpful/supportive	Engaging and communicative	Role model/mentor	Kind and polite
Helpful Supportive Supporting other academics Supporting with assessments Cooperative Encouraging Collaborative Pastoral care Help increase confidence	Engaging Communicative Good communicator Good at explaining Interactive Able to teach well Able to explain well Passion for teaching Enthusiastic	Role model Mentor Motivator Guide Inspiring Guidance on careers, research and clinical placements Invaluable Essential	Kind Polite Benevolent Empathy Attentive Caring Patient

Educator/facilitator	Clinically experienced/Professional	Knowledgeable	Strong work ethic
Educator Facilitator Academic guide Educational support Class admin Versatile/flexible	Clinically experienced Professional Teaching clinical application Teaching clinical skills	Knowledgeable Confident Insightful Intelligent Sharp	Thorough Organised Hardworking Up to date

Friendly and approachable	Near peer	Leader	Career advisor
Friendly Approachable Easy going Open	Near peer	Leader	Career advisor

The Top 3 attributes, expressed as those with a higher frequency of response, were helpful/supportive, friendly/approachable and educator/facilitator. Students most valued helpful/supportive (24.2%), while staff prioritised educator/facilitator (32.6%). CTFs equally valued being friendly/approachable, engaging and communicative and educator/facilitator (20% each). Table 2 shows the breakdown of attributes among CTFs, staff and students.

**TABLE 2:** Frequency of CTFs' attributes perceived by students, CTFs and staff. Crosstabulation [*χ* ^2^(1,*n* = 364) = 43.26, *p* 0.004, phi = 0.345].

	Student attributes (*n* = 306)	CTF attributes (*n* = 15)	Staff attributes (*n* = 43)	Total attributes (*n* = 364)
Helpful/supportive	74 (24.2%)	0	12 (27.9%)	86 (23.6%)
Friendly and approachable	52 (17%)	3 (20%)	2 (4.7%)	57 (15.7%)
Educator/facilitator	30 (9.8%)	3 (20%)	14 (32.6%)	47 (12.9%)
Engaging and communicative	41 (13.4%)	3 (20%)	2 (4.7%)	46 (12.6%)
Knowledgeable	27 (8.8%)	1 (6.7%)	2 (4.7%)	30 (8.2%)
Role model	22 (7.2%)	1 (6.7%)	6 (14%)	29 (8%)
Kind and polite	26 (8.5%)	1 (6.7%)	2 (4.7%)	29 (8%)
Near‐peer	9 (2.9%)	1 (6.7%)	1 (2.3%)	11 (3%)
Clinically experienced/professional	9 (2.9%)	1 (6.7%)	0	10 (2.7%)
Career advisor	8 (2.6%)	0	0	8 (2.6%)
Leader	6 (2%)	0	0	6 (1.6%)
Strong work ethic	2 (0.7%)	1 (6.7%)	2 (4.7%)	5 (1.4%)

### 3.2 Analysis of CTF Contribution to Students' Learning Experiences

The open‐ended survey responses (see Table 3) highlighted that CTFs contribute to students' learning by combining teaching expertise with pastoral care. Both students and staff emphasised CTFs' roles in clarifying the curriculum, providing clinical context and being approachable role models. CTFs also recognised the importance of being accessible and relevant to inspire students to persist in their studies.

**TABLE 3:** Thematic map and illustrative quotes from the open‐ended question ‘How do CTFs contribute to the quality of the students' learning experience?’

Theme	Subtheme	Quotes from students	Quotes from CTFs	Quotes from staff
Teaching expertise	CTFs contribute to academic support/facilitation	*Guiding us to good understanding of the topics covered in tutorials … able to answer any queries we may have about any topics (Student 15)*	*Facilitating the development of applying acquired knowledge in both self‐directed and collaborative situations (CTF 3)*	*They play a vital role in delivering tutorials, lectures and most of the assessment related activities (Staff 6)*
	CTFs integrate clinical context into science learning	*Allow us to bridge the gap between pre‐clinical knowledge and clinically applying our learning (Students 11,30)* *They provide clinical experience and help to link theory to practice. (Students 3,32,34,59)*	*Providing clinical context to student learning (CTF 1)*	*They help to integrate the basic science knowledge within a clinical context (Staff 12)*
Pastoral care	CTFs act as role model/mentor to inspire and support students	*They share their own experiences with us (Students 3,35,49)* *They like to give advice about future career (Student 51)*	*Acting as a role model to inspire students to persevere with studying medicine (CTF 1)*	*They provide mentoring and are a role model for the students to aspire to (Staff 9)*
	CTFs are approachable and act as near peer support for students	*Because the CTFs are less senior/more near peer than more senior staff they are easier to speak to and approach with problems (Students 19,95)* *They're relatable since a lot of them are close in age to us so we feel more comfortable in approaching them (Student 22)* *Ctfs are great at drawing on their experience and informing students of recommendations/things they regret to allow med student to have a better experience of med school. (Students 29,57)*		*They have recent knowledge of what it is like being a medical student. Students can relate better to a CTF (Staff 14)*
	CTFs are accessible and supportive	*They are very good not only in supporting us from an academic point of view, but we also get support from a pastoral point of view when we need it (Student 54)*	*We are often the first port of call for student queries and support (CTF 2)*	*They provide support (Staff 8)*

### 3.3 Analysis of Focus Groups

The focus groups centred on discussing the survey themes, elaborating on the Top 3 attributes identified in the survey analysis (see Table 4).

**TABLE 4:** Thematic map and illustrative quotes from the focus groups, when participants were asked ‘What do you think is the role of a CTF?’

Theme	Subtheme	Quotes from students	Quotes from CTFs	Quotes from staff
Educator/facilitator	CTFs provide academic support and guidance	*They act as teachers, facilitate the tutorials. They are like a channel between the block leads and the students (Student 1)*	*Our role was to explore student's knowledge and realise their own strength and weaknesses because we meant to chat to them and not actually tell them things, but get them to realise where they need to read up or what they need to do (CTF 3)*	*Of all the things they do the major thing is that they teach (Staff 7)* *Support for the educational team (Staff 8)*
	CTFs integrate clinical context into learning	*During the basic sciences years, so years one and two, it's more about linking all the basic science concepts that we have got from the lectures to their clinical application. It's been important to bring the clinical context into what we learn. (Students 7,8)*	*Providing students with clinical context for the learning of the basic sciences (CTF 2)*	*I think the role of a clinical teaching fellow is really to try to get them to understand the importance of topics that they are learning by providing real life examples in clinical practice (Staff 6)*
Helpful and supportive	CTFs are aspirational role models	*Were also role model to look at them and feeling like ‘ohh I want to be like this person’ (Student 11)* *They been in my shoes before and, another thing as well was sort of like discussing my future aspirations with them and receiving sort like of guidance (Student 1)*		*They're invaluable as role models for how we want our students to be going forwards (Staff 4)*
	CTFs provide real life career advice	*Even general questions about how the Med school works, about our Futures Foundation years, specialty training, all those things (Student 7)* *Was sort of like discussing my future aspirations with them and receiving sort like of guidance (Student 1)* *We can have conversations about our career plans, about our experiences at medical school, sharing research, others ideas much more accurately as compared to the lecturers (Student 7)*	*One of the attributes being more like career guidance because the 1st and 2nd years have a lot of questions, they do not really know how a lot of things work, when it comes to being a doctor and kind of the progression (CTF 1)* *When they get a bit more comfortable with you and they are asking you more, not really personal stuff, but more pastoral stuff about, sort of career advice (CTF 6)*	*Sort of show the students why they have chosen the speciality they have, ‘I chose this speciality because I love this bit and this bit and this bit’ and I think that really adds something to the role and the credibility at the students' sides (Staff 4)*
	CTFs share insightful stories and experiences	*We had this one session on infection with the lecturer where the CTF was alongside the lecturer and we were getting their insight about what happened during hospital, what kind of infections they encountered in. That was really useful (Student 2)* *It was nice to hear some of their stories, especially when they linked to some of the clinical cases. I thought it just brought everything. It tied everything in really nicely (Student 9)*		*Students place a lot of trust in the CTFs. I think because they see them so often and the CTFS have got a lot of recent and relevant stories (Staff 6)* *When they are working with the students, they often will tell them something from their experience clinically. And that's so useful. It really reinforces for the students and makes it more real for the students as well. So, you know, when we are going through the group work questions and it's basic biomedical science, but the CTFs can say ‘Ohh yeah, I saw a case of this’ and then talk about it and that really brings it home I think for the students (Staff 2)*
	CTFs provide pastoral support	*Supporting the students, we can go to them, discuss anything that's bothering us (Student 7)*	*Pastoral support because we see them quite frequently (CTF 3)*	*I think that's very powerful for the students to have a young teaching fellow as their tutor cause they can really discuss some things that medical students might struggle with. I do think that's quite important, that pastoral role that they play (Staff 4)*
Friendly and approachable	CTFs are near‐peer and relatable	*It's basically like having a medical teacher to allow us to know what resources we need to use and how we need to learn from someone who has already done it and was gone through these stages (Student 12)* *Because they just graduated from medical school, they are quite young, they can relate to us easier (Student 10)* *With CTF they are a bit closer to our career paths … So I think because of that reason we develop closer bonds with the CTFS (Student 7)*	*To act as a near peer. Which I'm thinking about like having done exams recently, been in Med school, so being like a near peer to the students (CTF 2)*	*CTFs are near‐peers …*. I *think that with a younger person it's easier to build that sort of safe space when you are practicing (Staff 4)*
	CTFs build connections and foster sense of community	*It's not always just related to Tutorial, but could be ‘How is your day’, for example, in that sort where there is no academic … I think they make the learning process more personal (Student 3)* *With CTF they are a bit closer to our career paths and because we have more encounter with them in a much more one to one way compared to the lectures … So I think because of that reason we develop closer bonds with the CTFS (Student 7)*	.*.building a relationship with students. (CTF1)* *Building connection with students you know over a period of time has been really nice. The ones that you know their name and you know you have built some form of connection relationship with them over the year. I'd like to say that we'd keep connection with some of the students if I'm honest. I'm not sure how realistic that is. I mean, I'm going to be working in hospitals that they'll be placed in, so it's likely I'll see them (CTF 2)*	*They can form a better lesson with CTFs, maybe because CTFS are also potentially a little bit younger and are also more often with them. So they develop a very nice relation to where they feel much more comfortable to discuss and ask and not be silent (Staff 7)*
	CTFs are valued members of the education team	*I felt more comfortable asking questions from the CTFS then anyone else. I think they are very approachable*. *I think the CTFS are all really kind people. They are not annoyed by a lot of questions (Student 8)* *They really care that we understand the content, so they take time to explain everything (Student 8)*	*I had friendly slash, approachable personality (CTF 4)* *A good communicator. And committed to teaching. Ohh and enjoyment teaching (CTF 4)*	*Patience because you know you have to be fairly patient especially with the first years when they are new to the whole thing (Staff 2)* *CTFs are very friendly, very flexible, very supportive (Staff 8)* *A good communicator which all our CTF seem to be very good and also empathy (Staff 1)* *CTFs always seemed to be really nice and helpful and engaging (Staff 5)*

## 4 Discussion

Our study sought to explore the perceptions of early years medical students regarding the role of CTFs and how these aligned with CTFs' and academics' views of the same role. Our findings highlight the unique role of CTFs in early years of medical education, who work closely with medical students, offering mentorship and pastoral support that extends traditional teaching responsibilities. This university‐based model offers several advantages. First, CTFs provide continuous academic and pastoral support, addressing challenges and promoting a positive learning environment. Second, they facilitate the early integration of clinical context by connecting basic science with clinical relevance, helping students bridge the gap between theory and practice, making learning more meaningful and applicable. Finally, CTFs help foster a sense of trust and belonging by offering consistent support from familiar educators, thereby building a strong sense of community and continuity throughout the early stages of medical training.

This university‐based model offers several advantages. First, CTFs provide continuous academic and pastoral support.

Our study reveals varying perspectives on CTFs' contributions. CTFs offer more than just teaching; they shape students' aspirations, provide mentorship and create a supportive learning environment. This is facilitated by CTFs often being closer in age to students than academic staff and having the shared experience of recently going through medical school, both of which can improve relatability and rapport between student and teacher. Near‐peer literature supports the idea that near‐peer educators can play a crucial role in students' personal and professional development [11, 12]. Students appreciated the approachability of CTFs, which facilitated open communication and reduced the intimidation often felt with senior educators [6].

This is facilitated by CTFs often being closer in age to students than academic staff and having the shared experience of recently going through medical school.

In contrast, staff members prioritised the CTFs' role as ‘educator/facilitator’, in accordance with general indications that faculty members place more attention on academic objectives [21]. Staff perceived the CTFs to significantly enhance the students' learning experience by integrating clinical context into basic sciences through sharing real‐life clinical experiences. Interestingly, CTFs did not prioritise clinical context integration while students viewed it as an added benefit to role modelling. Role modelling was also perceived by students and staff as offering advice on career progression. This integration is critical in the early years of medical education, where students are beginning to form connections between their academic learning and future clinical practice [14]. CTFs attributed their opportunity for building connections to the frequent and regular interaction with students [22].

Final year students have been shown to view CTFs as role models [8], but this has not yet been shown in early year students. Our findings align with Harden and Crosby's [23] roles of a medical educator, where mentorship and role modelling were crucial but underappreciated. Interestingly, CTFs also prioritised their clinical and academic roles; however, in time, there was increased awareness of their value in supporting and mentoring early year students.

CTFs also prioritised their clinical and academic roles; however, in time, there was increased awareness of their value in supporting and mentoring early year students.

The pastoral care provided by CTFs was another crucial aspect highlighted by both students and staff. While technical attributes like ‘knowledgeable’ and ‘having a strong work ethic’ were important, personal qualities were more highly regarded by students. This suggests that students in the early years value the CTF‐learner relationship and look at them as role models, which is crucial as medical education increasingly emphasises a humanistic approach to patient care [24]. The pastoral role is particularly important in the early pre‐clinical years of a medical education programme, where students may experience stress [15] and imposter syndrome when moving into the clinical years [25]. These feelings could be the consequence of low confidence of their preparedness for clinical training and its extensive practical content [26, 27].

### 4.1 Implications for Medical Schools

Our findings have several implications for structuring CTF roles in medical schools. Mentoring helps to build relationships with students and positively influences student outcomes, including a sense of belonging and self‐confidence in professional skills and abilities [21]. Mentoring offered to students in the clinical years has shown to have a positive impact on their well‐being and personal growth, helping to deal with the challenges that lie ahead when becoming doctors. This is even more effective when the mentor is closer to the level of the mentee and not someone who is higher up the organisational hierarchy [28]. As students value relational qualities over academic expertise in early years, medical schools should provide training to enhance these mentoring and pastoral care skills. This would foster a more supportive environment, especially during the transition to clinical placements [16].

This is even more effective when the mentor is closer to the level of the mentee and not someone who is higher up the organisational hierarchy.

### 4.2 Limitations and Future Research

This study was conducted in a single UK medical school, limiting generalizability of the findings. However, single‐site studies provide an opportunity for in‐depth analysis and control over variables, which is valuable for foundational research [29]. Future research should explore the role of CTFs in the early years across multiple institutions and conduct longitudinal studies examining the long‐term impact on students' transition to the clinical years.

## 5 Conclusion

The role of CTFs is multifaceted and should be tailored to the specific needs of the year groups they interact with. Students in the early years of medical education place significant value on the mentoring and supportive skills that CTFs offer, prioritising these over their academic and clinical expertise. Medical schools should formalise opportunities for CTFs to develop these skills and ensure that academic staff support these extended roles. Further research is needed to develop an early years‐focused CTF model that could serve as a blueprint for medical institutions. Such a model would aim to provide continuous and focused support, thereby ensuring a seamless transition for students into their clinical years.

Medical schools should formalise opportunities for CTFs to develop these skills and ensure that academic staff support these extended roles.

## Author Contributions

**Nadia Lascar:** conceptualization, methodology, data curation, investigation, writing – original draft, validation, visualization, writing – review and editing, formal analysis, project administration. **Wendy Leadbeater:** methodology, investigation, writing – original draft, writing – review and editing, formal analysis. **Noor Al‐Antary:** investigation, writing – review and editing. **Joseph Cowling:** investigation, writing – review and editing. **Claire Joanne Stocker:** methodology, validation, formal analysis, supervision, writing – review and editing.

## Ethics Statement

This study was reviewed and approved May 2022 by the Health and Life Sciences Ethics Committee, Aston University reference #1827.

## Conflicts of Interest

The authors declare no conflicts of interest.

## Supporting information


**Appendix S1.** Supporting Information.

## Data Availability

Data available on request from the authors.
