# Supplementary material for: Prioritising Mentorship: The Key Attributes We Should Focus on From Our Clinical Teaching Fellows in Early Years Medical Education
Source: Clin Teach. 2025 Feb 21;22(2):e70053. doi: 10.1111/tct.70053 (PMC11843849; doi:10.1111/tct.70053)
Supplement: Supplementary file 1 — Appendix S1. Supporting Information. [file TCT-22-e70053-s001.docx]

**APPENDIX 1. Focus group recruitment and interview guide**

Recruitment Process

Following the completion of the survey, participants were invited to join focus groups to delve deeper into their responses. All CTFs, staff, and students who completed the survey were asked if they wished to participate in the focus groups. Recruitment aimed to achieve small group sizes for rich, interactive discussions. A total of 12 students (split into two groups), 8 staff members (two groups), and 6 CTFs participated.

Purpose of Focus Groups

These discussions aimed to expand on survey findings, offering comprehensive insights into the perceptions of the CTFs’ role and the attributes identified in the survey analysis. Participants individually reviewed and coded responses for similarities, contrasts, beneficial topics, and significant points before collaboratively identifying the main themes emerging from the data.

Focus Group Guide Development

An interview guide containing open-ended questions was developed, informed by established methods (e.g., Legard et al., 2003; Krueger, 2000). This guide encouraged participants to articulate their views and experiences while allowing the moderator to explore key aspects in depth. The guide was refined after being tested with colleagues and adjusted further during the first few sessions to ensure clarity and relevance.

Focus Group Procedure

During the sessions, the lead researcher acted as the moderator, beginning discussions by probing participants’ experiences and encouraging dialogue on agreement or disagreement. Key research questions were explored in detail (Breen, 2006). Prompts and follow-up questions, based on Kvale (1996), facilitated in-depth responses, clarified participant meanings, and encouraged elaboration. Sessions concluded with the question: “Is there anything you would like to add?” This ensured that participants could discuss any unaddressed topics.

Ethical Considerations

Participants were informed about the privacy policy, anonymity, and recording procedures, and signed consent forms. Clear rules for group discussions were outlined at the start of each session.

Focus Group Topics and Structure

A summary of the focus group structure and topics is provided in Table 1.

**Table 1. Focus group guide**

| **Introduction (10 min)**   - Welcome and thank participants. - Introduce the research team and participants. - Provide a brief overview of the research project. - Obtain informed consent and explain privacy and anonymity policies. - Outline focus group rules and answer any initial questions.   **Discussion topics (45 min)**   1. **Opening Question**   What do you think is the role of a clinical teaching fellow?  Discuss survey-identified attributes.   1. **Introductory Question**   Staff: What is your experience of working with clinical teaching fellows?  CTFs: What is your experience of working as a clinical teaching fellow?  Students: What is your experience of learning with clinical teaching fellows?   1. **Transition Question**   Staff: What aspects do you value of working with clinical teaching fellows?  CTFs: What aspects do you enjoy of working as a clinical teaching fellow?  Students: What aspects do you value of learning with clinical teaching fellows?   1. **Key Questions**   Staff: How have CTFs supported your teaching activities?  CTFs: How have you supported activities led by other academic staff?  Students: How have CTFs supported and provided feedback during your learning activities?  All groups: How do CTFs contribute to the quality of students’ learning experiences?   1. **Ending Question**   Is there anything about the role of clinical teaching fellows that we haven’t discussed and you would like to address?  **Conclusion (5 min)**   - Summary of the key points emerged during the discussion - Close the focus group and thank the interviewees for their participation |
| --- |
|  |

**References**

1. Krueger, R. (2000) Focus Groups: A Practical Guide for Applied Research (London: Sage).
2. Legard, R., J. Keegan and K. Ward (2003). In-depth interviews. Qualitative Research Practice. J. Ritchie and J. Lewis. London, Sage: 138-169.
3. Rosanna L. Breen (2006) A Practical Guide to Focus-Group Research, Journal of Geography in Higher Education, 30:3, 463-475.
